# The alternative *Medicago truncatula* defense proteome of ROS—defective transgenic roots during early microbial infection

**DOI:** 10.3389/fpls.2014.00341

**Published:** 2014-07-17

**Authors:** Leonard M. Kiirika, Udo Schmitz, Frank Colditz

**Affiliations:** Department of Plant Molecular Biology, Institute of Plant Genetics, Leibniz University HannoverHannover, Germany

**Keywords:** Gene silencing, GTPase ROP9, *Medicago truncatula*, pathogenic interactions, ROS—reactive oxygen species, RNA interference, symbiotic interactions, suppression of ROS

## Abstract

ROP-type GTPases of plants function as molecular switches within elementary signal transduction pathways such as the regulation of ROS synthesis via activation of NADPH oxidases (RBOH-respiratory burst oxidase homolog in plants). Previously, we reported that silencing of the *Medicago truncatula* GTPase MtROP9 led to reduced ROS production and suppressed induction of ROS-related enzymes in transgenic roots (MtROP9i) infected with pathogenic (*Aphanomyces euteiches*) and symbiotic microorganisms (*Glomus intraradices*, *Sinorhizobium meliloti*). While fungal infections were enhanced, *S. meliloti* infection was drastically impaired. In this study, we investigate the temporal proteome response of *M. truncatula* MtROP9i transgenic roots during the same microbial interactions under conditions of deprived potential to synthesize ROS. In comparison with control roots (Mtvector), we present a comprehensive proteomic analysis using sensitive MS protein identification. For four early infection time-points (1, 3, 5, 24 hpi), 733 spots were found to be different in abundance: 213 spots comprising 984 proteins (607 unique) were identified after *S. meliloti* infection, 230 spots comprising 796 proteins (580 unique) after *G. intraradices* infection, and 290 spots comprising 1240 proteins (828 unique) after *A. euteiches* infection. Data evaluation by GelMap in combination with a heatmap tool allowed recognition of key proteome changes during microbial interactions under conditions of hampered ROS synthesis. Overall, the number of induced proteins in MtROP9i was low as compared with controls, indicating a dual function of ROS in defense signaling as well as alternative response patterns activated during microbial infection. Qualitative analysis of induced proteins showed that enzymes linked to ROS production and scavenging were highly induced in control roots, while in MtROP9i the majority of proteins were involved in alternative defense pathways such as cell wall and protein degradation.

## Introduction

RAC/ROP (Rho of plants) are plant-specific small GTPases that function as simple binary molecular switches within elementary signal transduction pathways by cycling between GTP-bound on modes and GDP-bound off modes. In the GTP-bound forms, they interact with specific downstream effectors, mediating a wide repertoire of molecular stimuli that provoke cellular responses (Poraty-Gavra et al., 2013). ROPs integrate many upstream signals via the guanine nucleotide exchange factors (GEFs), guanine nucleotide dissociation inhibitors (GDIs) and GTPase-activating proteins (GAPs), regulating downstream effectors such as Rop-interactive CRIB motif-containing proteins (RICs) and interactor of constitutively active ROPs (ICRs) (Nagawa et al., 2010). Small GTPases are well studied in mammals and yeast cells and have been grouped into various subfamilies depending on their functional properties (Schiene et al., 2000). ROPs are known to function in different developmental processes including polarized cell growth, pollen tube and root hair development, hormonal signaling as well as cell morphogenesis (Yang and Fu, 2007; Liu et al., 2010). They are also implicated in regulating several cellular processes including vesicle trafficking, cytoskeleton organization and dynamics, auxin transport and response to pathogens (Nibau et al., 2006; Yalovsky et al., 2008; Lorek et al., 2010; Wu et al., 2011; Poraty-Gavra et al., 2013). The ICRs have also been shown to regulate polarized secretion and polar transport of auxin during auxin-regulated development. ROP proteins form key regulatory elements for reactive oxygen species (ROS) generation in plant cells especially at the plasma membrane by activating the NADH oxidases termed as RBOH (for respiratory burst oxidase homolog). In *Medicago truncatula*, 16 putative ROPs were suggested from assembled EST sequences (Yuksel and Memon, 2008) but only seven ROPs have been confirmed (Liu et al., 2010) with the rest either being artifacts or redundant. In *Arabidopsis thaliana,* 11 ROPs are identified (Winge et al., 1997).

During the initial microbial invasion, the host plant confers a general defense reaction characterized by rapid activation of a wide repertoire of symbiotic or pathogenic defense cellular responses. More often, the ROS, especially H_2_O_2_, form the hallmark of these very early host defense systems that cause a hypersensitive reaction culminating in host cell death at the site of infection (Puppo et al., 2013). ROS are also said to diffuse across the cell membranes via the aquaporins and function as second messenger during signal transduction pathway hence acting as elementary signal molecules for activation of plant defense responses (Borisova et al., 2012). The superoxide and hydrogen peroxide are the typical ROS accumulated in host cells. The delicate balance between production and scavenging activity allows the duality in function of ROS to exist in plant system orchestrated by a large network of enzymes and antioxidative compounds. The scavenging activity of ROS in the mitochondria is controlled by the alternative oxidase (AOX), non-proton-pumping, alternative type II and the Ca^2+^-dependent NADPH dehydrogenase (Steffens et al., 2013). The antioxidative activity in the cell is provided by molecules such as glutathione, tocopherols, tannins, phenolic compounds and ROS scavenging enzymes such as superoxide dismutase (SOD), ascorbate peroxidase, glutathione peroxidase, catalase as well as other non-enzymatic proteins such as metallothioneins and thioredoxin that lead to ROS homeostasis (Steffens et al., 2013). The ROS scavenging mechanisms lead to cell wall structural reinforcement by cross-linking of various extracellular proteins including proline-rich glycoproteins to the polysaccharide matrix (Djébali et al., 2011). In rice (*Oryza sativa*), the GTPase OsRac1 was shown to positively regulate disease resistance by stimulating the NADPH-mediated ROS production via direct binding to the catalytic subunit of NADPH oxidase N-terminal extension specific for RBOH proteins (Kawasaki et al., 2006; Jones et al., 2007; Nakashima et al., 2008). *In vivo* fluorescence resonance energy transfer (FRET) analysis showed that the Ca^2+^ concentration in the cytosol may regulate the RBOH-Rac interaction, hence modulating the activity of NADPH oxidase in ROS production (Wong et al., 2007).

Legumes (Fabaceae) interact with soil-borne microbes (Colditz and Braun, 2010) and are unique in establishing symbiosis with rhizobia bacteria, which ultimately leads to nitrogen fixation in the formed structures known as nodules. Through their association also with arbuscular mycorrhizal fungi, legumes benefit by acquiring macronutrients including phosphorus and nitrogen, as well as most likely an array of micronutrients in exchange for up to 20% of the plant-fixed carbon (Finlay, 2008). ROPs are shown to play a key role during rhizobia infection in the process of nodule development and also during the establishment of mycorrhizal association (Berken, 2006). Expression of MtROP3, MtROP5 and MtROP6 in *M. truncatula* increased after rhizobia inoculation as reported by Liu et al. (2010), while in *L. japonicus*, the LjROP6 was shown to act as a positive regulator of infection thread formation during rhizobia infection (Ke et al., 2012).

Previously, we reported that silencing of MtROP9 impairs rhizobial infection but positively regulates root colonization by arbuscular mycorrizal fungi *G. intraradices* and oomycete pathogen *A. euteiches* (Kiirika et al., 2012). The infection process in MtROP9i transgenic roots was characterized by clear reduction of ROS accumulation in the cells, and by marked transcriptional suppression of ROS-related enzymes such as RBOH and catalase. Both symbiotic and pathogenic interactions are known to induce oxidative burst coupled with induction of defense-related products such as PR proteins, where the difference in the two forms of interactions is suggested to be of quantitative in nature especially with regard to ROS production. In addition, the generation of ROS suppresses the expression of PR genes (Peleg-Grossman et al., 2012). In *M. truncatula*, the MtSpk1 gene encoding a putative protein kinase was induced by exogenous application of H_2_O_2_ as well as nodulation factor indicating the functional role of ROS in regulating genes directly linked to rhizobia symbiosis (Andrio et al., 2013). Transient decrease in MtRBOHs gene expression was reported to lead to decrease in ROS efflux observed 1 h after *M. truncatula* roots treated with NF (Lohar et al., 2007).

In this study, we have utilized the previously investigated sequence of MsRac1 sequence from *Medicago sativa* for RNA interference (RNAi)-mediated gene silencing in the model legume *M. truncatula* where we identified a *M. truncatula* sequence ortholog annotated as MtROP9 (TC173331; Dana-Farber Cancer Institute *M. truncatula* Gene Index [MtGI]; Quackenbush et al., 2001). A gene-specific region of MtROP9 was selected for RNAi gene knockdown with *Agrobacterium rhizogenes* used as a vector. Evaluation of MtROP9i root proteome maps via 2D IEF SDS-PAGE and MS after symbiotic and pathogenic interactions at the very early timepoints of infections revealed changes in protein profiles as clearly visualized using the heatmap-GelMap tool.

## Materials and methods

### Construction of RNAi vector

Transgenic MtROP9i roots were produced as reported previously (Kiirika et al., 2012), using the binary vector pK7GWIWG2(II)::DsRED (kindly provided by E. Limpens; Limpens et al., 2005) containing the gene for red fluorescent marker DsRED1. The vector was modified by insertion of two sequence cassettes (in the sense-antisense direction) encoding parts of the putative effector (G2) and GTPase (G3) domains of the MsRac1 ortholog MtROP9 (TC173331; GenBank accession no. AF498359). Binary vectors for gene knockdown by RNAi were constructed using the Gateway technology (Invitrogen Life Technologies). Gene-specific oligonucleotides (ROP9attb1_for, 5′-attB1 GTGTTACTGTTGGTGATG-3′;ROP9attb2_rev,5′-attB2-ACGCCTTCACGTTCTCC-3′) with attached attB adapters were obtained from the *Medicago sativa* MsRac1 sequence (GenBank accession no. AJ251210; Schiene et al., 2000). Using these oligonucleotides, amplification of a 461-bp fragment from *Medicago truncatula* cDNA was carried out and the PCR products purified using the QIAquick PCR purification kit (Qiagen) and cloned into the pDONR221 donor vector (Invitrogen). In the second cloning step, the inserts were transferred into the Gateway-compatible binary vectors mentioned above followed by transformation into *Agrobacterium rhizogenes* strain ArquaI (Quandt et al., 1993) using standard methods. The presence of the MtROP9 sequence fragments and the sense-antisense orientation of the cloned fragments in the T-DNA were confirmed by sequencing the construct, while non-modified binary vectors were transformed into *A. rhizogenes* ArquaI as a control.

### Generation of transgenic roots and infection assays

*M. truncatula* MtROP9i and Mtvector transgenic roots were generated according to the Boisson-Dernier et al. (2001) using *M. truncatula* (Jemalong A17) wild-type plantlets as described before (Colditz et al., 2007). *M. truncatula* composite plants with roots transformed by *A. rhizogenes* were cultured stably on M medium (Bécard and Fortin, 1988) containing 25 mg L21 kanamycin for selection and decreasing concentrations of 350 to 0 mg L21 ticarcillin disodium/clavulanate potassium (Duchefa) to stop growth of *A. rhizogenes*. Twelve individual populations of composite plants with MtROP9i and Mtvector transgenic roots were generated independently via *A. rhizogenes* transformation, containing at least 200 plants each. Populations were cultured on M medium and kept in the growth chambers at 22°C, 65% humidity, 16-h photoperiod at 220 μE m^−2^ s^−1^. Inoculation with *A. euteiches* (ATCC 201684) was carried out as described before (Colditz et al., 2007). Each transgenic root population was infected with 500 mL of lake water containing 250,000 *A. euteiches* vital zoospores. Inoculation with *G. intraradices* was performed using commercially available inoculums (Granular AMF inoculum; BIORIZE). Infection with *Sinorhizobium meliloti* wild-type strain Rm2011 was performed as described previously (Schenkluhn et al., 2010).

### Protein isolation, 2D IEF SDS-PAGE and gel evaluation

Phenol extraction of total protein from the cells was carried out as described previously (Colditz et al., 2004, 2005, 2007). For IEF, 3 mg of protein was diluted with 350 μ l of rehydration buffer, consisting of 8 M urea, 2% (wt/vol) CHAPS, 100 mM dithiothreitol, 0.5% (vol/vol) IPG buffer for correspondent p*I* range (pH 3–11 non-linear [NL]; Amersham Pharmacia Biotech, Uppsala, Sweden), and a trace of bromphenol blue. 2D IEF SDS-PAGE was performed for the three infections and at four timepoints of harvesting by combining the IEF strips (IPGphor system) with a sodium dodecyl sulfate–tricine gel electrophoresis (Protean II XL, 20 by 20 cm; BioRad, Richmond, CA, U.S.A.) as already described (Colditz et al., 2005). Gels were stained with 0.1% (wt/vol) Coomassie Brilliant Blue (BioRad) overnight and scanned on a UMAX Power Look III Scanner (UMAX Technologies, Fremont, CA, U.S.A.). Gels were evaluated using Delta 2D software, version 4.0 (Decodon, Greifswald, Germany) with three replicates per group (1, 3, 5, and 24 hpi). Spots detection was done automatically and occasionally corrected manually. In gel normalization was performed using the Delta 2D software for the overlays of three replicate gels each. Spots with a relative spot volume of less than 0.05% were deleted and the significant abundance of spots between MtROP9i and Mtvector groups was determined using Student's *t*-test (confidence interval ≥95%) based on the relative spot volume.

### Visual evaluation of protein induction patterns via gelmap extended by a heatmap tool

To explicitly present the voluminous protein dataset comprising of all significantly induced proteins from the three infections, i.e., *S. meliloti*, *G. intraradices* and *A. euteiches* in both transgenic root populations MtROP9i and Mtvector, the novel software GelMap (http://www.gelmap.de) which is utilized for protein annotation was used (Klodmann et al., 2011; Senkler and Braun, 2012). In addition, a heatmap tool was integrated to the GelMap module allowing a clear visualization of induction patterns of all identified proteins according to differences in abundance. This technique utilizes the inbuilt function filters in the GelMap software for any user-defined cluster of choice, based on specific physiological functions of proteins. The presented Heatmap (**Figure 2**; https://gelmap.de/532) was created by clustering all protein induction values of proteins of similar physiological functions both for major and sub-categories found at a certain selected infection time-point. The total inductions per sub-category presented as individual values were automatically filtered and displayed on a matrix. The Heatmap was then generated by applying a color-coding system which visually indicates the range of protein induction for a certain (sub-) category from dark-red to light-red, corresponding to high protein inductions and low protein inductions, respectively. Proteins with the highest levels of induction either in MtROP9i or Mtvector were selected or ranked first as the most predominant physiological categories in the Heatmap-GelMap (**Table 2**).

### Mass spectrometry and creation of heatmap-gelmap

Protein spots of 1.4 mm diameter were cut from Coomassie stained gels using a GelPal Protein Excision manual spot picker (Genetix, Great Britain) and in-gel digested with Trypsin as described by Klodmann et al. (2011). Tryptic peptides were further analyzed by nanoHPLC (Proxeon, Thermo Scientific) coupled to electrospray ionization quadrupole time of flight MS (micrOQTOF Q II, Bruker Daltonics), using all settings and parameters as described previously (Klodmann et al., 2011). Data processing and protein identification was carried out with ProteinScape 2.0 (Bruker Daltonics) and the MASCOT search engine querying three *Medicago*-specific protein databases [*Mt3.5 ProteinSeq, NCBI Medicago truncatula protein, and Mtf*(*asta*)2] available at the LegProt db (Lei et al., 2011) as well as *SwissProt*, using the following parameters: trypsin/P; one missed cleavage allowed; fixed modifications: carbamidomethylation (C), variable modifications: acetylation (N) and oxidation (M); precursor ion mass tolerance, 30 ppm; peptide score >24; charges 1C, 2C, 3C. Protein and peptide assessments with MASCOT scores above 25 were considered. Heatmaps were created using the total significant induction (≥1.5 fold) of proteins per physiological function as annotated via *Swissprot*. Induction of proteins with similar functions was clustered and presented as values, which were filtered and displayed on a matrix. Color-coding scheme ranging from dark red (high induction) to light red (low induction) was applied. A Heatmap image (.jpg) was assigned *x*- and *y*-coordinates to specifically allocate total inductions of each protein category into a corresponding color-code range on a Heatmap matrix (**Figure 2**). The Heatmap was linked with the reference GelMap platform (Senkler and Braun, 2012). An Excel (Microsoft) file containing all protein information including MS/MS results and the corresponding Heatmap image (.jpg) were then imported into GelMap portal. Information on GelMap creation can be accessed at http://www.gelmap.de/howto. The heatmap tool integrated to the GelMap, as utilized for protein annotation (Klodmann et al., 2011), allowed explicit presentation of voluminous dataset and clear visualization of induction patterns of all identified proteins according to differences in abundance. This technique utilizes the inbuilt function filters in the GelMap software for any user-defined cluster of choice, based on specific physiological functions of proteins. It clusters all protein induction values of proteins of similar physiological functions both for major and sub-categories found at a certain selected infection time-point.

## Results and discussion

### Experimental setup

The aim of this study was to evaluate the temporal proteome response of *M. truncatula* roots to symbiotic and pathogenic infection under conditions of significantly suppressed potential for the legume host to synthesize ROS as a common and early defense mechanism. We generated transgenic *M. truncatula* (Jemalong A17) root populations expressing an RNAi hairpin construct for the RAC-type GTPase MtROP9 (TC173331; MtGI at Dana-Farber Cancer Institute; Quackenbush et al., 2001) as described and characterized by us previously (Kiirika et al., 2012). The transgenic roots termed MtROP9i revealed significantly reduced levels of ROS production as compared to transgenic empty vector control (Mtvector) roots (Kiirika et al., 2012). As a consequence, MtROP9i roots exhibited clearly altered infection levels when inoculated with symbiotic and pathogenic microbes (Kiirika et al., 2012).

**Figure 1:**
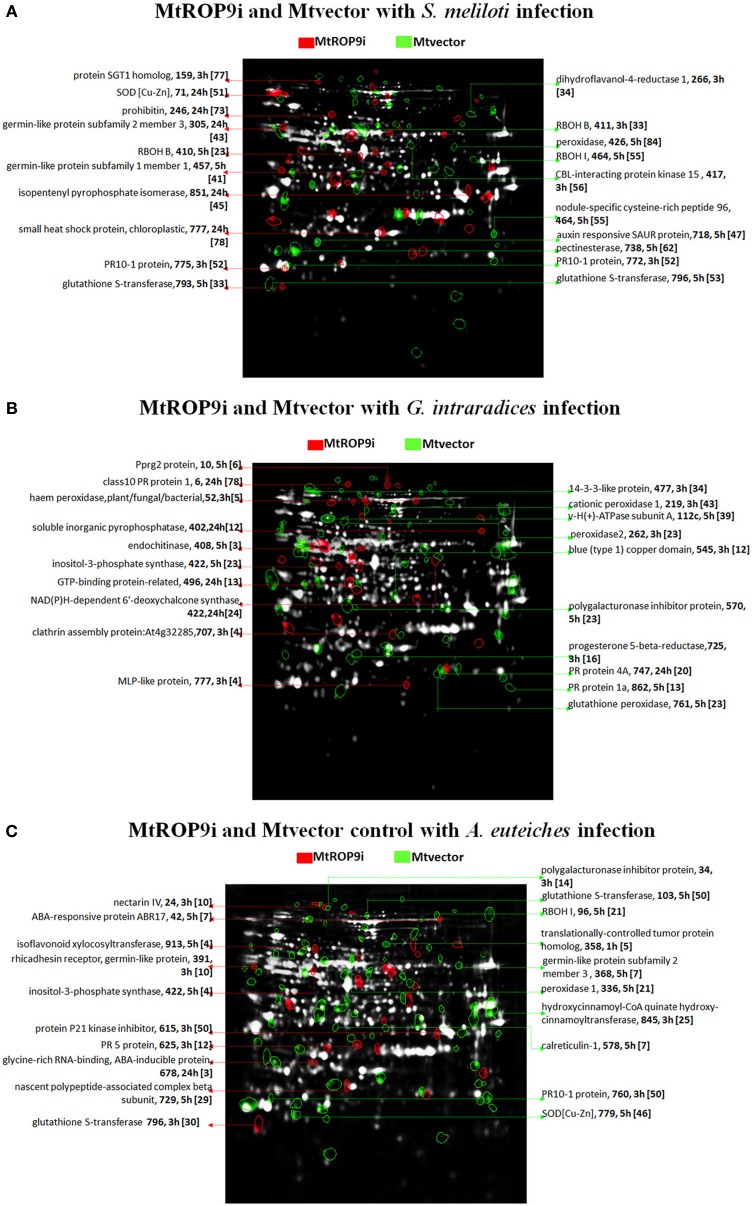
**Proteins of high abundance in MtROP9i (red spots) and Mtvector (green spots) after *S. meliloti* (A), *G. intraradices* (B) and *A. euteiches* (C) infections for the predominant physiological categories defense response, stress response, signal transduction, secondary metabolite biosynthesis and transport.** Proteins of equal abundance appear as white spots. The protein name, spot number, hours post inoculation (h) is given per protein as well as the level of abundance based on >1.5-fold, shown in square brackets.

Twelve individual populations of MtROP9i were constructed independently via *A. rhizogenes*-mediated root transformation, each representing at least 200 stably growing composite plants with transgenic roots. For our infection assays and proteomic analyses, only MtROP9i and Mtvector roots comprising on average 60% and more of transformed (transformation marker/*Ds*RED-positive) roots were considered. For validation of the effective knockdown in MtROP9i transgenic roots, MtROP9 transcript abundance was determined by reverse transcription (RT)-quantitative PCR as previously described (Kiirika et al., 2012). The relative expression of MtROP9 was drastically reduced about >90% in these root populations and did not increase after microbial infection when compared with Mtvector roots (data not shown). Importantly, MtROP9i transgenic roots of all considered populations revealed no significant ROS production nor its accumulation after infections with the chosen compatible root microbes as confirmed via *in planta* luminometric ROS assays while Mtvector roots did (data not shown). Inoculation assays were conducted using rhizobial bacterium *S. meliloti* as well as arbuscular mycorrhizal fungus *G. intraradices* for symbiotic interactions, and the legume root pathogen *A. euteiches* (oomycota). The microbial inoculations resulted in similar infection patterns for MtROP9i and Mtvector transgenic roots as previously described (Kiirika et al., 2012) and thus are not explicitly shown here. For the protein analyses, proteins from four independent isolations and from four different time-points of harvest (1, 3, 5, and 24 hpi) were separated using two-dimensional (2-D) IEF/SDS PAGE. Three of the proteome maps achieved per single investigation were selected for Delta 2D analysis in order to decipher protein spots with significantly differential abundance (Figure 1). In parallel, proteins of interest revealing differences in abundance were excised from the gels for tryptic digestion and MS-based protein identification.

### Proteomic profiling of MtROP9i and Mtvector transgenic roots during symbiotic and pathogenic interactions

To identify changes in the proteomes of *M. truncatula* MtROP9i transgenic roots infected with *S. meliloti*, *G. intraradices* and *A. euteiches*, proteome maps of four early infection points (1 h, 3 h, 5 h, and 24 h) were prepared. Three Coomassie-stained gels were produced of infected root tissue of three individual MtROP9i and Mtvector populations for each time-point. These gels were evaluated by the Delta2D (v4.4) software (Decodon GmbH, Greifswald, Germany). Using student's *t*-test with a confidence interval ≥95%, significant changes in spot pattern depicted as differences in spot abundance (>1.5 fold) for different infections were determined based on the spot volume. These spots were selected for further MS analyses. For this purpose, the spots were manually excised from the Coomassie-stained gels, then in-gel digested with Trypsin and analyzed via nLC ESI-MS. Protein identification was carried out based on the *Medicago*-specific protein databases from the LegProt db (Lei et al., 2011), which allowed high rates of protein identification. The protein search tool ProteinsScape 2.0 (Bruker Daltonics) and MASCOT search engine were used for querying the three Medicago-specific databases [*Mt3.5 ProteinSeq, NCBI Medicago truncatula protein* and *Mtf*(*asta*)^2^] as well as *Swissprot*.

By following this procedure, in total 733 spots were identified to be different in abundance at the four considered time-points in gels of infected MtROP9i roots as compared to control roots. 213 spots from *S. meliloti* infections were found with different abundances, representing a total of 984 MS-identified proteins that comprised 607 unique proteins. From the 984 proteins, 385 and 568 proteins were identified in MtROP9i and the control, respectively. A comparable number of 230 spots of differences in abundance were found in gels for the *G. intraradices* infections, whereof 796 proteins were identified revealing a total of 580 unique proteins. From these 796 proteins, 311 and 485 proteins were induced in MtROP9i and the control, respectively. Infections with *A. euteiches* revealed 290 spots with different abundances after the software-based evaluation, giving an increased number of 1240 proteins in total, whereof 828 were unique proteins. 1240 proteins consist of 456 and 784 proteins induced in MtROP9i and the control, respectively, after pathogenic infection.

Overall, the total number of induced proteins identified in MtROP9i transgenic roots after microbial infections was found to be lower as compared to the protein number in Mtvector. This can be attributed to the knock-down of the signaling protein MtROP9 which is directly involved in several early signaling cascades. However, the highest individual protein induction levels were found in Mtvector roots infected with *S. meliloti* as compared to fungal infections with *A. euteiches* and *G. intraradices* (Figure 1; Tables 1, 2).

**Table 1:** **Protein categories induced only in specific infections**.

**Physiological function (main category)**	**Physiological function (sub-category)**
***SINORHIZOBIUM MELILOTI***	
Secondary metabolite biosynthesis	Aromatic compounds biosynthesis
Signal transduction	Hormone metabolism (auxin responsive SAUR protein)
	Nodulation
Nucleic acid metabolism	Pyrimidine biosynthesis
Energy metabolism	Sulfur metabolism
	Pyruvate and TCA cycle metabolism (malate dehydrogenase)
***APHANOMYCES EUTEICHES***	
Defense response	Chaperone activity
	Protease inhibitor (kunitz-type)
	Proteolysis
Ion binding & cofactor activity	Oxidoreductase activity
Lipid/fatty acid metabolism	Lipid & fatty acid biosynthesis
Protein folding & processing	Protein modification
	RNA binding
Signal transduction	Protein translocation
Stress response.	Hormone metabolism
***GLOMUS INTRARADICES***	
Protein folding and processing	Protein modification
Signal transduction	Protein-protein interaction(14-3-3-like protein)
	Ion binding(blue (Type 1) copper domain)
	Intracellular components binding (Ran binding protein)
Stress response	Heat shock protein (stromal 70 kDa heat shock-related protein)
Energy metabolism	Oxidative phosphorylation (ATP synthase subunit delta)
Lipid/fatty acid metabolism	Lipid & fatty acid biosynthesis
Amino acid metabolism	NAD binding
Transport	Protein transport

List of physiological function categories (main and sub-categories) of all proteins identified exclusively upon specific infections with S. meliloti, A. euteiches and G. intraradices.

**Table 2:**
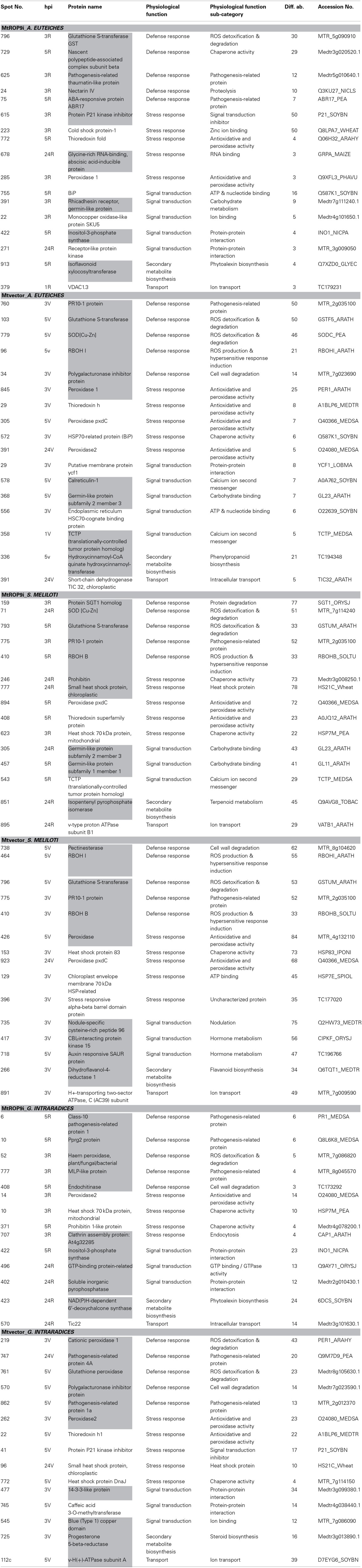
**List of proteins with highest induction in abundance for selected physiological categories**.

List of proteins identified with highest induction in abundance for the selected physiological function categories after infection with S. meliloti, A. euteiches and G. intraradices for the time points 1 h, 3 h, 5 h, and 24 h. Proteins names that are underlayed with gray color are discussed in the manuscript.

Considering the induction pattern along a timeline for the three infections, protein induction was high at the early points of infection in Mtvector with majority of proteins reaching maximum induction even at 3 hpi as compared to MtROP9i. In MtROP9i, low protein induction at the early infection time-points much likely indicates the absence of the first line of defense comprising of ROS defense signaling. Thus, early cellular defense reactions were affected by the silencing of the signaling protein MtROP9. In addition, also the majority of other alternatively induced enzymes which are not directly related to ROS signaling such as those involved in cell wall and protein degradation as well as PR proteins were highly induced especially during the advanced stages of infection.

### Categorization of induced proteins in MtROP9i and control

All identified proteins were further classified according to their physiological functions and ordered based on the most predominant physiological categories, with four categories representing the majority of induced proteins: (i) defense response, (ii) stress response, (iii) signal transduction, and (iv) secondary metabolite biosynthesis.

In Figure 1, proteins with the highest differential abundance in each infection were filtered and visualized on a two-colored gel image channel. Evaluation of protein abundances revealed suppressed induction of ROS-related enzymes in MtROP9i transgenic roots as compared to Mtvector roots. In this category, 18 and 43 proteins were differentially induced in MtROP9i and Mtvector, respectively, after *S. meliloti* infection, while 29 and 42 proteins were induced in MtROP9i and Mtvector, respectively in *G. intraradices* infection. In *A. euteiches* infection, 31 and 69 proteins were induced in MtROP9i and Mtvector, respectively. These proteins are intuitively linked to ROS production, hypersensitive response reaction as well as enzymes responsible for ROS scavenging. They include RBOH1 (55 fold at 5 h) and peroxidase (84 fold at 5 h) induced after *S. meliloti* infection in Mtvector as compared to MtROP9i roots. SOD [Cu-Zn] (46 fold at 5 h), RBOH1(21 fold at 5 h), peroxidase 1 (25 fold at 3 h) and peroxidase pxdc (7 fold at 5 h) were induced after *A. euteiches* infection as well as cationic peroxidase 1 (43 fold at 3 h), glutathione peroxidase (23 fold at 5 h), peroxidase 2 (23 fold at 3 h) and thioredoxin h1 (22 fold at 5 h) after *G. intraradices* infection in Mtvector compared to MtROP9i transgenic roots.

Proteins that were found be highest in abundance after *S. meliloti* infection include SGT1 homolog (77 fold at 3 h), prohibitin (73 fold at 24 h), germin-like protein subfamily 2 member 3 (43-fold at 24 h) and isopentenyl pyrophosphate isomerase (45 fold at 24 h) in MtROP9i roots, while pectinesterase (62 fold at 5 h), peroxidase (84 fold at 5 h), nodule-specific cysteine-rich peptide 96 (78 fold at 3 h) and dihydroflavanol-4-reductase 1 (34 fold at 3 h) were found in Mtvector roots (Table 2). In *G. intraradices* infections, PR10-1 (6 fold at 5 h), inosito-3-phosphate synthase (23 fold at 5 h) and NAD(P)H-dependent 6'-deoxychalcone synthase (24 fold at 24 h) were identified in MtROP9i roots while 14-3-3-like protein (34 fold at 3 h), cationic peroxidase 1 (43 fold at 3 h) and progesterone 5-beta-reductase (16 fold at 3 h) in Mtvector roots. In *A. euteiches* infection, protein P21 kinase inhibitor (50 fold at 3 h), isoflavonoid xylocosyltransferase (4 fold at 5 h), and ricadhesin receptor, germin-like protein (9 fold at 3 h) were identified in MtROP9i while PR10-1 protein (50 fold 3 h), peroxidase 1(25 fold at 3 h), and hydroxycinnamoyl-CoA quinate hydroxy-cinnamoyltransferase (21 fold at 5 h) in Mtvector roots (Figure 1; Table 2).

### Overview of protein induction via heatmap

**Figure 2:**
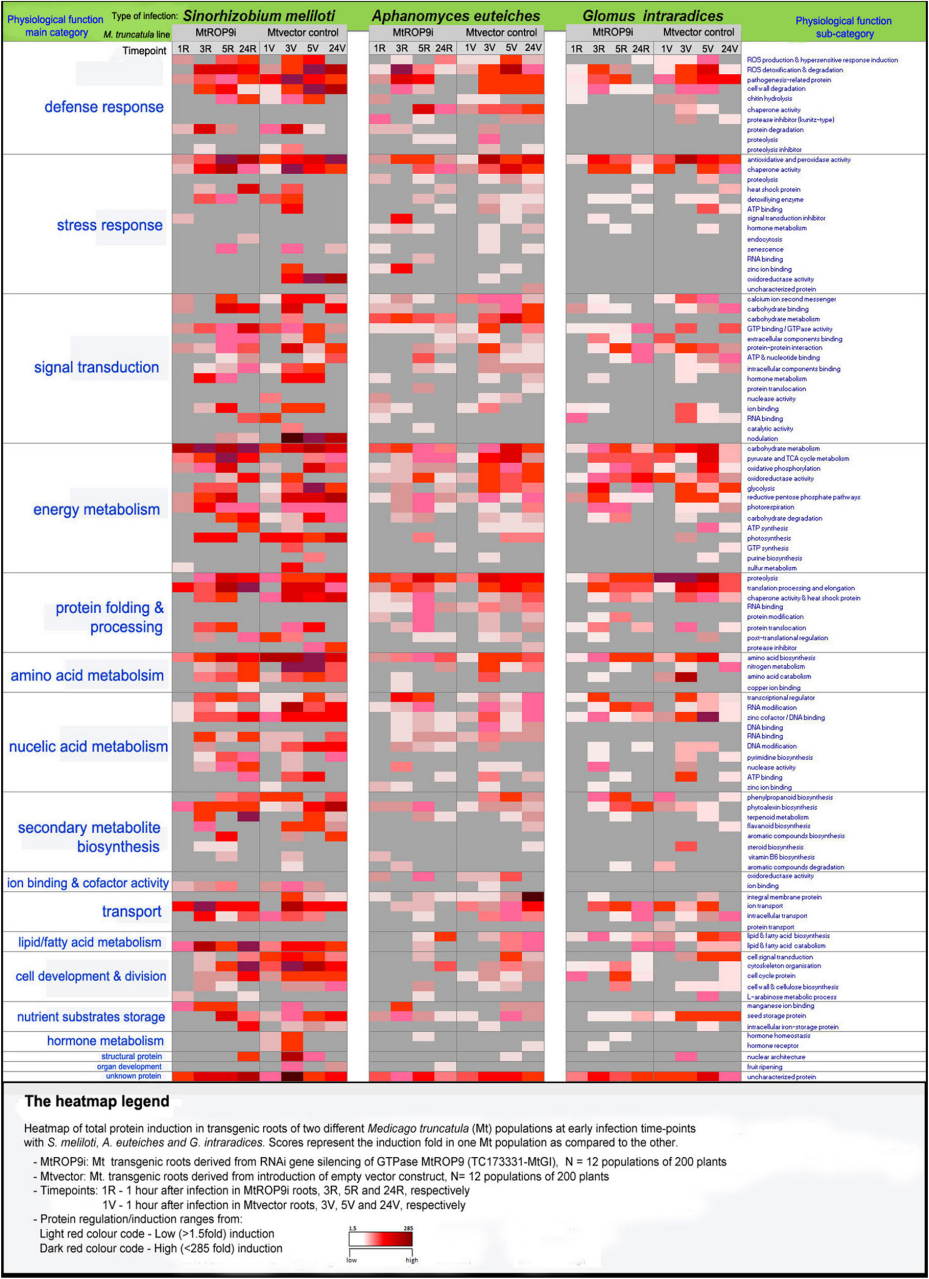
**The heatmap legend**.

To explicitly present the voluminous protein dataset comprising of all significantly induced proteins from the three infections applied in both transgenic root populations MtROP9i and Mtvector, the novel online software GelMap (http://www.gelmap.de) which is utilized for protein annotation was used (Klodmann et al., 2011; Senkler and Braun, 2012). In addition, a novel *Heatmap tool* was integrated to the GelMap module allowing a clear visualization of induction patterns of all identified proteins according to differences in abundance. The presented Heatmap (Figure 2; https://gelmap.de/532) was created by clustering all protein induction values of proteins of similar physiological functions both for major and sub-categories found at a certain selected infection time-point.

Overall, a comparative analysis of induced protein patterns considering all infections showed a distinctively lower number of total proteins induced in MtROP9i transgenic roots both for pathogenic and symbiotic infections as compared to Mtvector roots (Figure 2; Supplementary Figures 1A–C, 2A–C). This indicates the responsive role of ROP GTPase MtROP9 protein during early microbial infection signaling. Hereby - under conditions of hampered ROS synthesis—induction of early defense-related infection protein network mainly comprised of ROS-related enzymes was significantly affected in MtROP9i roots, forming the first line of plant host defense (Figure 2; Supplementary Figures 1A–C). The amount of total protein induction during rhizobial infection was found significantly higher as compared to the two fungal infections investigated. Considering fungal infections, the induction pattern reached maximum at 5 hpi in control roots with significant reduction at 24 hpi, but with rhizobia infection, the induction reached maximum as early as at 3 hpi remaining constant even at a later timepoint (Supplementary Figures 1A–C: minigraphs; Supplementary Figures 2A–C).

Visual evaluation of this Heatmap-GelMap for microbial infections in MtROP9i and Mtvector led to detection of major results as exemplarily summarized in the following:

- The total number of induced proteins identified in MtROP9i transgenic roots after symbiotic and pathogenic infections were significantly lower as compared to control roots, indicating that the infection proteome network was affected after silencing the signaling protein MtROP9 and therewith the perpetual suppression of ROS defense signaling. Interestingly, the individual protein inductions following rhizobia infection with *S. meliloti* was the highest in control roots as compared to fungal infections with pathogenic oomycete *A. euteiches* and symbiotic interactions with *G. intraradices*. The high protein inductions in rhizobia infections could be attributed to the high specificity of *S. meliloti*—*M. truncatula* infection and genomic adaptation of the host plant, but also to the similarities between rhizobial bacteria and pathogenic bacteria as shown via phylogenetic analyses based on their sequences (Willems and Collins, 1993; Young et al., 2011). In addition, several other proteins have been induced to promote formation of rhizobia symbiosis such as the nodule-specific cysteine-rich peptide 96, dihydroflavanol-4-reductase 1. However, the total number of individual proteins induced in *A. euteiches* infections was higher, where it can be surmised that the host in addition to induction of the early ROS defense signaling enzymes engages other alternative pathways particularly at the advanced stages of infection process, including induction of cell wall degrading enzymes such as polygalacturonase inhibitor, pectinesterase and SGT1 protein as well as PR proteins.
- Induction of proteins involved in ROS production and detoxification was significantly reduced in MtROP9i roots as compared to control roots after microbial infection (Supplementary Figures 2A–C). RBOH 1 and RBOH B were highly induced at the early points of *S. meliloti* infection particularly in control roots but clearly reduced in MtROP9i. In addition, their induction levels considering *G. intraradices* and *A. euteiches* infections were moderate in control roots. SOD [Cu-Zn] was highly induced after *S. meliloti* infection, but its induction was noted only for the three timepoints 3, 5, and 24 hpi. The decline in induction of proteins responsible for ROS production and the concomitant decrease of detoxifying enzymes at 12 hpi is indicative of the essential role of ROS during root and nodule development (Lohar et al., 2007; Puppo et al., 2013). A transient decline in ROS activity is thought to be essential for early NF signaling and the infection process at the root hairs (Lohar et al., 2007). In contrast, permanently suppressed levels of ROS due to inactive RBOH proteins as documented for MtROP9i transgenic roots led to a drastic impairment of early rhizobial infection accompanied by abnormal root hair deformations (Kiirika et al., 2012). Regained accumulation of ROS after 12 hpi in control roots could have multiple functions, one as part of a typical defense response to limit bacteria entry, second, as compounds needed for progression of infection thread or as signals for symbiotic protein synthesis (Soto et al., 2011).
- 92 proteins, classified as stress-related proteins were highly induced after *S. meliloti* infection with 34 and 58 proteins identified in gels of MtROP9i and control roots, respectively. Among them, the peroxidases (84 fold at 5 h) involved in scavenging of peroxide and detoxification of ROS was highly induced. Several other enzymes including peroxidases, catalases, SOD (Cu-Zn) as well as enzymes involved in ROS production and hypersensitive response induction such as harpin binding protein 1, hydroxyacylxylotathione hydrolase as well as perforin domain containing protein 1 were also detected.
- 109 proteins involved in signal transduction were found to be highly induced where 33 and 76 proteins were induced in MtROP9i and controls, respectively. Calmodulin (CaM) 1, 2, 8 and CaM binding proteins were induced in the category of calcium second messengers, which constitutes proteins involved in the initial plant-microbe interactions. CaM mediates the calcium-dependent signaling by functioning as a decoder for the Ca^2+^ signatures during signal transduction especially at the early points of infection (Bender and Snedden, 2013). Induction of CaM depicts progressive colonization of the tissue by the pathogen thereby eliciting the secondary line of defense barrier. Calreticulin protein that functions in controlling plant defense by regulating the concentration of Ca^2+^ ions in the cell during signaling pathways as well as acting as molecular chaperone (Qiu et al., 2012) was induced only in control roots, particularly in fungal infections and not in MtROP9i. It was recently shown to confer resistance against oomycete pathogen phytopthora infestans in *Nicotiana benthamiana* (Matsukawa et al., 2013). Interestingly, the CaM and calreticulin proteins were highly induced in the Mtvector roots which also had concomitant high induction of ROS-related enzymes, suggesting a connection between ROS and calcium signatures. Both signals most likely represent a cross-talk that is constituted to modulate downstream nuclear activity resulting in induction of pathogenic or symbiotic specific protein networks.
- Proteins involved in antioxidative and peroxidase activity such as the thioredoxins were induced mainly at 3 and 5 hpi, especially after *A. euteiches* infection. The cytosolic form of thioredoxin h functions in response to oxidative stress which may have occurred at the early points of microbial infection, mainly pronounced in the control roots. Thioredoxin also accumulates in self-incompatibility reactions, seed germination and early seedling development (Chi et al., 2013).
- Paltry induction of ROS-related enzymes in MtROP9i transgenic roots after infection could not be ruled out especially after *S. meliloti* infection. This could be attributed to a knockdown but not a fully knockout of MtROP9 proteins or even the presence of other related GTP binding protein partners that as a consequence could potentially contribute to ROS synthesis at the cellular plasma membrane, even though at significantly reduced levels as compared to control roots leading to overall impairment of rhizobial infection signaling. Alternative proteins present in MtROP9i which belong to the category GTP binding/GTPase activity include the dynamin-related protein and guanine nucleotide-binding proteins.
- Proteins specific for rhizobia infection, such as the nodule-specific cysteine-rich peptide responsible for early nodulation signaling, were highly induced at 3 hpi after *S. meliloti* inoculation exclusively in control roots. The predominant induction of this protein pattern very well indicates the onset of rhizobial infection signaling in control roots. Since proteins involved in early infection signaling were not detected in MtROP9i roots, evidence for a hampered rhizobial infection is given as previously reported (Kiirika et al., 2012)
- G proteins have been shown to play a key role in early nodulation signaling (Choudhury and Pandey, 2013). Hence, low induction of proteins involved in nodulation in MtROP9i provides evidence to the interfered signal transduction process during root-rhizobia symbiosis due to *MtROP9* GTPase silencing as one of the major GTPases for early nodulation signaling.
- Major latex protein (MLP) and ABA-response protein (ABR17) related to the PR-10 proteins category were predominantly induced after *A. euteiches* and *G. intraradices* infections at 5 hpi in control as compared to MtROP9i. These proteins are induced when the intracellular ABA concentration increases in the cell during the course of infection. Generally, PR proteins comprising class 10, 1A, 5–1, and 1 as well as osmotin/thaumatin-like proteins were induced particularly in control roots infected with *S. meliloti*, indicating an introduction of a profound defense response also in rhizobial infections when ROS as signaling molecule is formed.
- Induction of PR proteins was previously reported both at the transcript and protein level (Colditz et al., 2004, 2005, 2007) forming the major components of molecular host defense response on *M. truncatula* after *A. euteiches* infection both at the early and later infection stages. The induction of PR proteins, in particular, was detected distinctively at advanced stages of *A. euteiches* infection (5 and 24 h) with predominantly high inductions in control roots as compared to MtROP9i roots. So far, reports indicate that 14 classes of PR protein are known in plants (Spoel and Dong, 2012). Previously, it was reported that especially both PR protein classes 5 and 10 were conjointly modified in expression following *A. euteiches* infections in *M. truncatula* (Colditz et al., 2007; Trapphoff et al., 2009). Several other proteins differentially induced were classified in the category of defense response, proteins involved in protein degradation, proteolysis and proteolysis inhibitor, induced only after *S. meliloti* and *A. euteiches.* Proteins involved in proteolysis, chaperone activity, protease inhibitor (kunitz-type) and protein modification were found induced exclusively after *A. euteiches* infection in control roots.
- In the category of secondary metabolite and biosynthesis, proteins responsible for phenylpropanoid biosynthesis (e.g., hydroxycinnamoyl-CoA quinate hydroxycinnamoyl-transferase), phytoalexin biosynthesis (e.g., chalcone-flavonone isomerase 1, isoflavonoid xylocosyltransferase and NAD(P)H-dependent 6'-deoxychalcone synthase) and flavonoid biosynthesis (e.g., dihydroflavanol-4-reductase 1 and CXE carboxylesterase) were found induced mainly in control roots.
- Structural protein (e.g., MFP1 attachment factor), hormone metabolism (e.g., abscisic acid receptor PYR1 and cytokinin-O-glucosyltransferase), cell division and development (e.g., actin, profiling and cyclin) and lipid/fatty acid metabolism (e.g., phospholipase D, fatty acid oxidation complex subunit alpha and lipoxygenase) were highly induced in control roots especially in Rhizobia infection as compared to fungal infections.
- Proteins playing key role in nodulation, sulfur metabolism and pyrimidine biosynthesis were only induced with *S. meliloti* infection in control roots.
- Proteins responsible for protein-protein interaction (14-3-3 like protein), ion binding (blue type copper domain) and NAD binding were only induced after *G. intraradices* infections (Table 1).

### Defense proteome of *M. truncatula* MtROP9i transgenic roots defective in ROS signaling

Of the 17 main physiological functional categories of induced proteins identified during the evaluations, significantly high numbers of proteins were found to be involved in defense response, stress response, signal transduction and energy metabolism.

During *S. meliloti* infections, 92 defense-related proteins were found to be induced with only low protein number (27 proteins) induced in MtROP9i as compared to control roots, where 65 proteins were induced. The majority of defense-related proteins induced in MtROP9i roots were those involved in alternative defense response pathways and not linked to ROS, indicating that the potential for host cell to synthesize ROS was compromised. Induction of protein SGT1 homolog (77 fold at 3 h) involved in protein degradation and the pathogenesis-related protein PR10-1 was found to be significantly high in MtROP9i roots as compared with Mtvector roots. Mitochondrial prohibitin (12 fold at 3 h) with the role of maintaining the integrity of the organelles was also significantly induced in MtROP9i. Recent findings shows that prohibitins are involved in mediating stress tolerance (abiotic stress, pathogen infection and elicitor signaling) as well as triggering retrograde signals in response to mitochondrial dysfunction (Aken et al., 2010).

Signaling proteins, such as proteins of the germin-like protein subfamily 1 and 2 (41 fold at 5 h and 45 fold at 24 h), which are carbohydrate binding proteins, were highly induced in MtROP9i roots. This indicates that early nodulation signaling is generally not affected by the gene silencing. Since the nodule-specific cysteine-rich peptide 96 (78 fold at 5 hpi) which plays a key role in the early infection signaling, was highly induced in control roots but not found in MtROP9i roots, it is much likely that infection processes shortly after the NF signaling are ROS-dependent via cross-linking of attachment proteins. Thus, in case of ROS depletion, lacking attachment of bacteria to root hairs results in impairment of rhizobial infection processes.

During *A. euteiches* infections, 165 proteins involved in defense response were identified, of which a significantly low number of proteins (64 proteins) were found induced in MtROP9i as compared to controls (101 proteins). Majority of these proteins induced in MtROP9i roots comprised those involved in alternative or secondary defense pathways giving an indication that the RBOH activity in ROS biosynthesis was affected. Cell wall degradation proteins including endochitinases (33 fold at 5 h) and polygalacturonase inhibitor protein (22 fold at 24 h), proteolysis such as nectarin IV (10 fold at 3 h), PR 5 protein (12 fold at 3 h) were highly induced in MtROP9i. PR-10-type proteins annotated as abscisic acid responsive proteins (AB17s) (7 fold at 5 h) were also induced in MtROP9i roots. Abscisic acid in *Arabidopsis* guard cells was shown to enhance levels of ROS (Pei et al., 2000). The meager traces of ROS-related enzymes detected at later stages of infection may have been enhanced by the presence of abscisic acid in the infected cells whereof indirectly affirmed by the significant induction of abscisic acid responsive proteins. Induction of PR proteins occurred at advanced stages of infection representing a secondary line of host defense system especially in MtROP9i roots. Their induction was also reported earlier with *A. euteiches* infections as the major component in the pathogen defense response of *M. truncatula* both at transcript and protein levels (Colditz et al., 2004, 2005, 2007; Schenkluhn et al., 2010).

Interestingly, a cytoplasmic redox protein non-expressor of PR1 (NPR1) was suppressed in MtROP9i roots and induced only in control roots at 5 hpi after pathogenic infection with *A. euteiches*, implying that its induction was hampered with the knockdown of MtROP9 protein. However, it was not induced in any of the symbiotic interactions. NPR1 protein functions in sensing salicylic acid (SA) during microbial infection where it exists in the cytoplasm as an oligomer in its non-induced forms but reduced to its monomeric form after pathogen infection and translocated to the nucleus leading to induction of PR proteins (Rochon et al., 2006; Vlot et al., 2009). Plants produce high concentrations of SA during systematic acquired resistance (SAR) both in infected and non-infected tissues that functions in the signaling of defense responses including induction of PR proteins (Oide et al., 2013). Presence of cytoplasmic ROS was shown to inhibit the action of NPR1 protein hence suppressing induction of PR proteins in *M. truncatula* seedlings infected with an incompatible bacteria *Pseudomonas syringae* (Peleg-Grossman et al., 2012). Based on our analyses, the suppressed induction of NPR1 protein at the early points of infections in MtROP9i but its particular induction in control roots could be due to high levels of ROS production in Mtvector.

The predominant induction of glutathione-S-transferase (GST) in control roots infected with *A. euteiches* with minimal induction in MtROP9i roots indicates the reduced activity of GST redox system responsible for primary antioxidative processes in the latter. The knockdown of MtROP9 also affected the expression of stress-related proteins. 51 proteins were induced in MtROP9i which was less compared with control roots where 81 proteins were induced after *A. euteiches* infection. Overall, 132 stress-related proteins were identified, which exhibited high inductions. Majority of stress-related proteins induced in MtROP9i roots were not linked to ROS production as found in control roots, but form part of other alternative cellular reaction pathways. The protein P21 kinase inhibitor, a potent cyclin-dependent kinase inhibitor (CDKI) was highly induced (50 fold at 3 h). P21 kinase inhibitor is induced as stress reaction of plants by deregulation of cell proliferation controlling growth pathways cell-cycle regulatory and integration of developmental signals in the cell machinery (De Veylder et al., 2001). A glycine-rich RNA-binding (3 fold at 24 h) as well as the cold shock protein-1(46 fold at 3 h) were induced in MtROP9i and are involved in post-transcriptional regulation of gene expression in plants under biotic or abiotic stresses (Mangeon et al., 2010).

Within *G. intraradices* infections, 43 defense-related proteins were induced where 15 and 28 proteins were induced in MtROP9i and control roots, respectively. PR proteins of class 10 (78 fold at 24 h) and Pprg2 (6 fold at 5 h) were induced in MtROP9i roots. The major latex protein (MLP) was detected both at 3 and 5 hpi. MLPs play a role in host defense (Betsy et al., 2009) and were only detected in fungal infections potraying their expression specificity to fungal infections. 92 stress-related proteins were highly induced after *G. intraradices* infection where 42 and 50 proteins were found in MtROP9i and control roots, respectively. Among the induced proteins in this category, clathrin assembly protein (At4g32285) (4 fold at 3 h), stress related proteins involved in the formation of vesicles in the cytoplasm for intracellular protein trafficking was highly induced in MtROP9i. Majority of highly induced proteins detected after *G. intraradices* infection in control roots comprised those specific for this type of infection, including the14-3-3-like protein (34 fold at 3 h), a family of signaling proteins, blue (type 1) copper protein (29 fold 5 h) involved in signal transduction as well as v-H(+)-ATPase subunit A (39 fold 5 h) playing a key role in membrane transport. 69 proteins involved in signal transduction were induced with 23 and 46 proteins induced in MtROP9i and control roots, respectively. The soluble inorganic pyrophosphate (12 fold at 24 h) involved in protein-protein interactions during signal transduction was highly induced in MtROP9i as compared with controls.

Several other proteins were differentially induced such as translationally-controlled tumor protein homolog (TCTP) exhibiting low induction in MtROP9i especially after *A. euteiches* infection as compared to control roots, which suggests the influence of MtROP9 knockdown. TCTP is shown to be a Ca^2+^ -binding protein ubiquitously expressed in all eukaryotic cells (Zhang et al., 2013). We reported its high induction at the transcript level in tomato treated with elicitors silicon and chitosan against soilborne bacteria *Ralstonia solanacearum* (Kiirika et al., 2013). Recent studies have also shown its involvement in negative regulation of hypersensitive reaction in *N. benthamiana* plants challenged with *R. solanacearum* (Gupta et al., 2013). Two modes of action of TCTP cytoprotective activity have been suggested, where it is said to act as a sequester of Ca^2+^ hampering programmed cell death (PCD) by reducing levels of Ca^2+^ in the cytosol or interact with other cytosolic membranous proteins of the cytosolic PCD machinery, thereby mitigating the progress in cell death. Caffeic acid 3-O-methyltransferase (CCOMT), a protein involved in conversion of caffeoyl-CoA to synapoyl-CoA, an intermediate in lignification in cells under stressed condition (Kosová et al., 2013) was induced particularly with the fungal infections while exhibiting low inductions in MtROP9i as compared to Mtvector roots. Taken together, the mechanisms of fungal invasion and ROS scavenging to the cell triggers CCOMT synthesis to function putatively as a compensatory response by enhancing lignin formation so as to reinforce the cell wall and maintain its functional integrity.

## Conclusions

The early molecular events in the cell upon microbial infection comprises ROS defense signaling as major first defense barrier. ROS generation is mainly related to activation of RBOH proteins which is achieved by protein interaction with cellular small GTPases, thus functioning as molecular switches. Therefore, from the current investigations, we have shown that silencing of small GTPase *MtROP9* in *M. truncatula* roots results in significant suppression of ROS-related enzymes at the early points of infection both for symbiotic and pathogenic interactions and a concomitant activation of alternative defense pathways by the host cell to counteract the infection progress. The induction of ROS-related enzymes such as RBOH 1/B, glutathione-S-transferase, peroxidases, catalases, SOD was found clearly suppressed in MtROP9i transgenic roots as compared to control roots. This implies that the RBOH activity responsible for oxidative burst during microbial infection was affected. Instead, proteins involved in alternative defense pathways such as cell wall degradation, chitin hydrolysis, proteolysis, and PR protein induction were highly induced. Other proteins whose induction was affected also were involved mainly in signal transduction pathways, general stress response, and chaperone activities. Since proteins involved in energy metabolism exhibited minimal differential induction in MtROP9i as compared with control roots, it clearly shows that ROS signaling is the major force for introduction of effective defense signaling, while enzymes related to energy metabolism which represents a prerequisite for a profound host infection defense generally remain retrievable. The total amount of protein induction was highest after *S. meliloti* infection in Mtvector, followed by the induction after pathogenic *A. euteiches* infection. Despite the overlapping similarities existing between pathogenic and symbiotic interactions, the host plant has to maintain specificity such that it activates response cues that are either meant to promote or desist the interaction. This process consists of broad array of protein networks forming central dynamics in the cell, which when analyzed give huge proteome datasets. The new Heatmap-GelMap module presented allowed systematic and easy visualization of all induced proteins displaying also their physiological functions in the cell. To this end, the elaborate protein induction evaluation upon symbiotic and pathogenic interactions reported herein, depicts the essential role of small GTPase MtROP9 during the early ROS defense signaling in plants.

### Conflict of interest statement

The authors declare that the research was conducted in the absence of any commercial or financial relationships that could be construed as a potential conflict of interest.

## Supplementary material

Supplementary Figure 1 **Protein induction patterns of *M. truncatula* MtROP9i and Mtvector roots after *S. meliloti* (A), *A. euteiches* (B) and *G. intraradices* (C) infection visualized on a Heatmap.** The heatmap shows distinct proteome subsets created from total induction values of all identified proteins classified into different physiological functions. The color intensity potrayed as a shift in gradient from light red to deep red represents the relative changes from a minimum to a maximum value allowing easy spotting of trends or standouts in each infection at a given time after infection. The total induction for each category is shown on the right and the minigraphs for the trend of total induction over 1, 3, 5, and 24 hpi is shown at the bottom.

Supplementary Figure 2 **Heatmap of total protein regulation in each physiological category over the four time-points for *S. meliloti* (A), *A. euteiches* (B) and *G. intraradices* (C) infections**.
