# Supplementary figures and images for: The alternative Medicago truncatula defense proteome of ROS—defective transgenic roots during early microbial infection
Source: Front Plant Sci. 2014 Jul 17;5:341. doi: 10.3389/fpls.2014.00341 (PMC4101433; doi:10.3389/fpls.2014.00341)

**Suppl. Fig. 1 (A) - *S. meliloti***

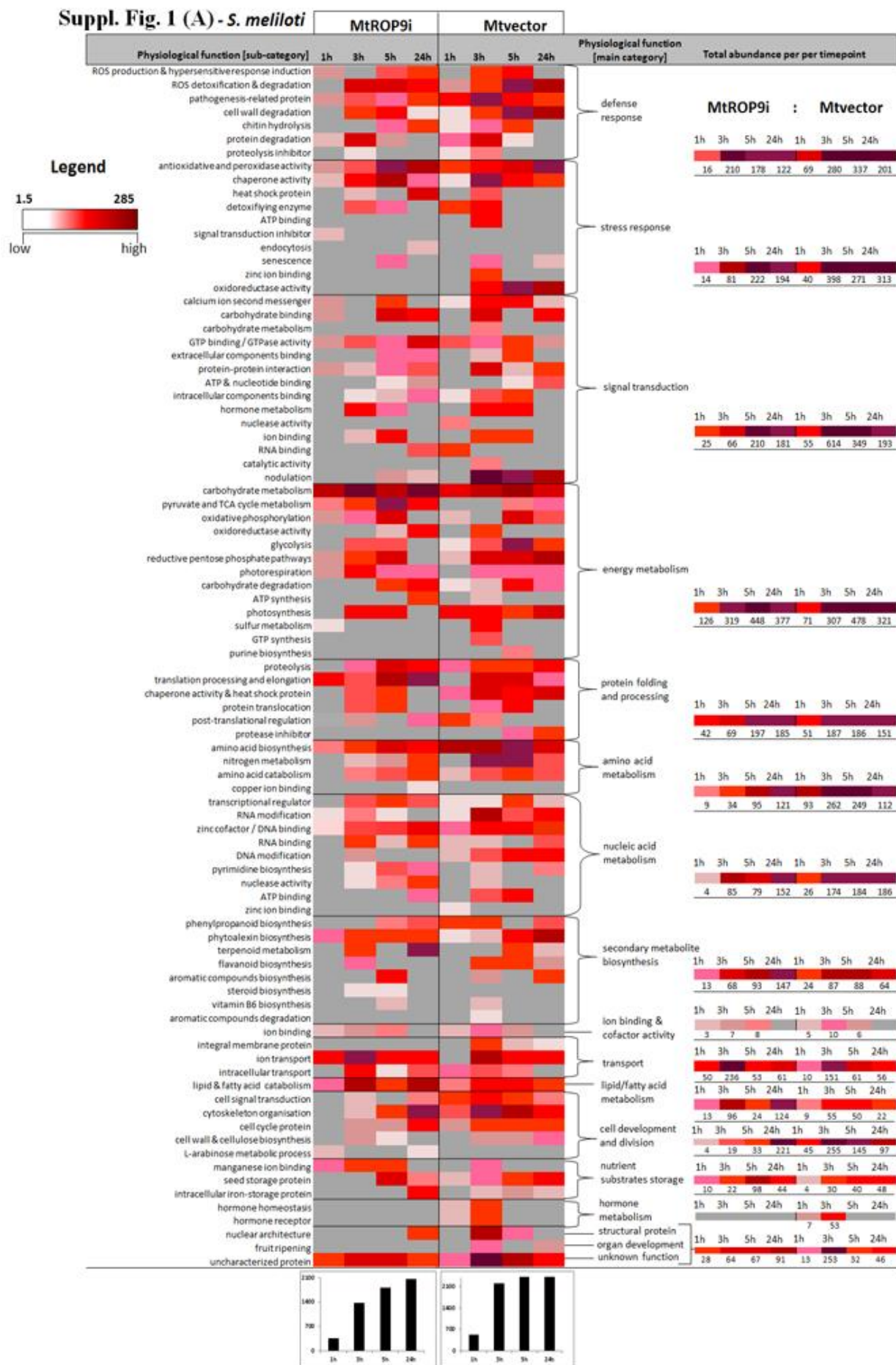

**Suppl. Fig. 1 (B) - *A. euteiches***

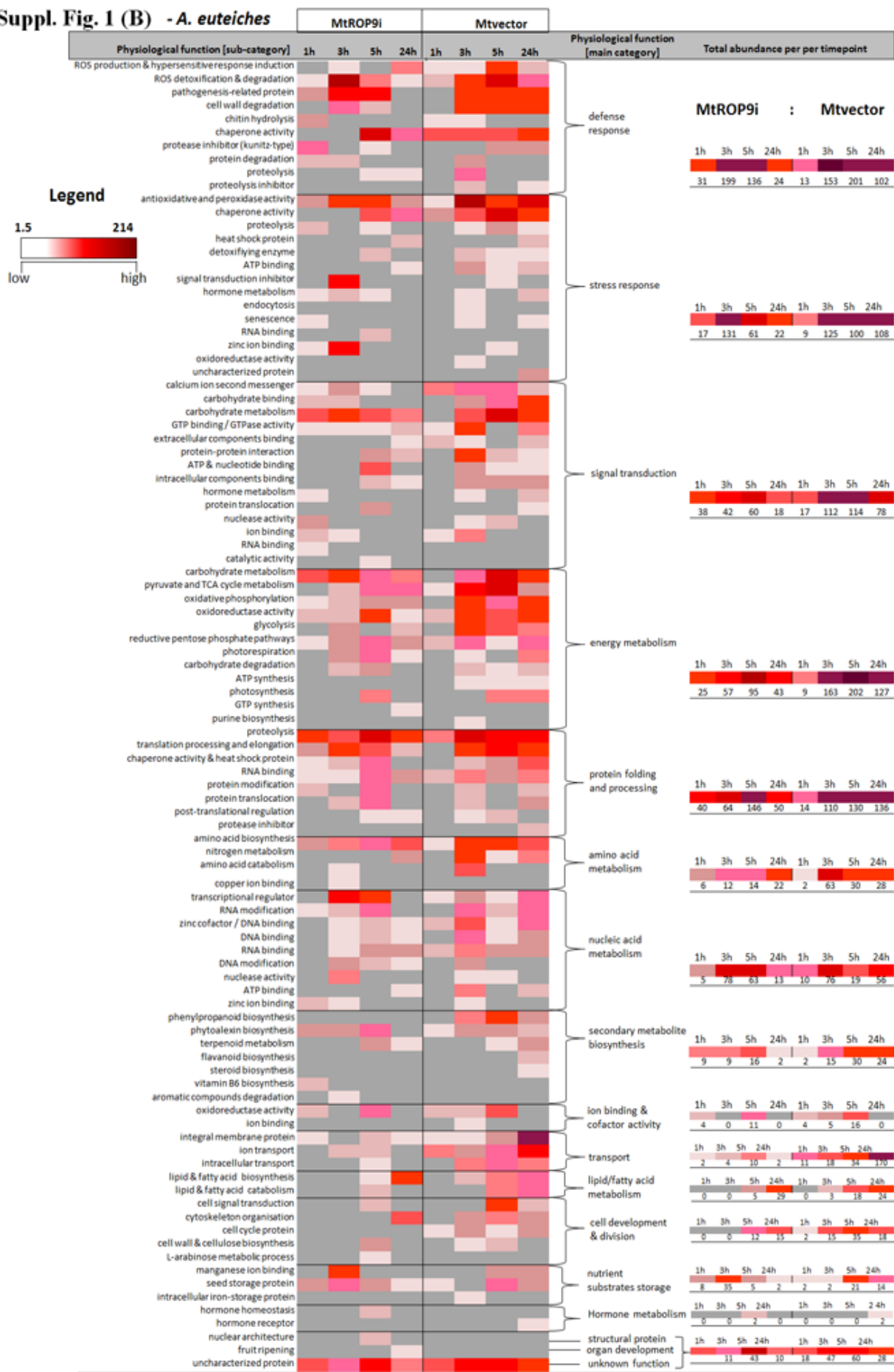

**Suppl. Fig. 1 (C) - *G. intraradices***

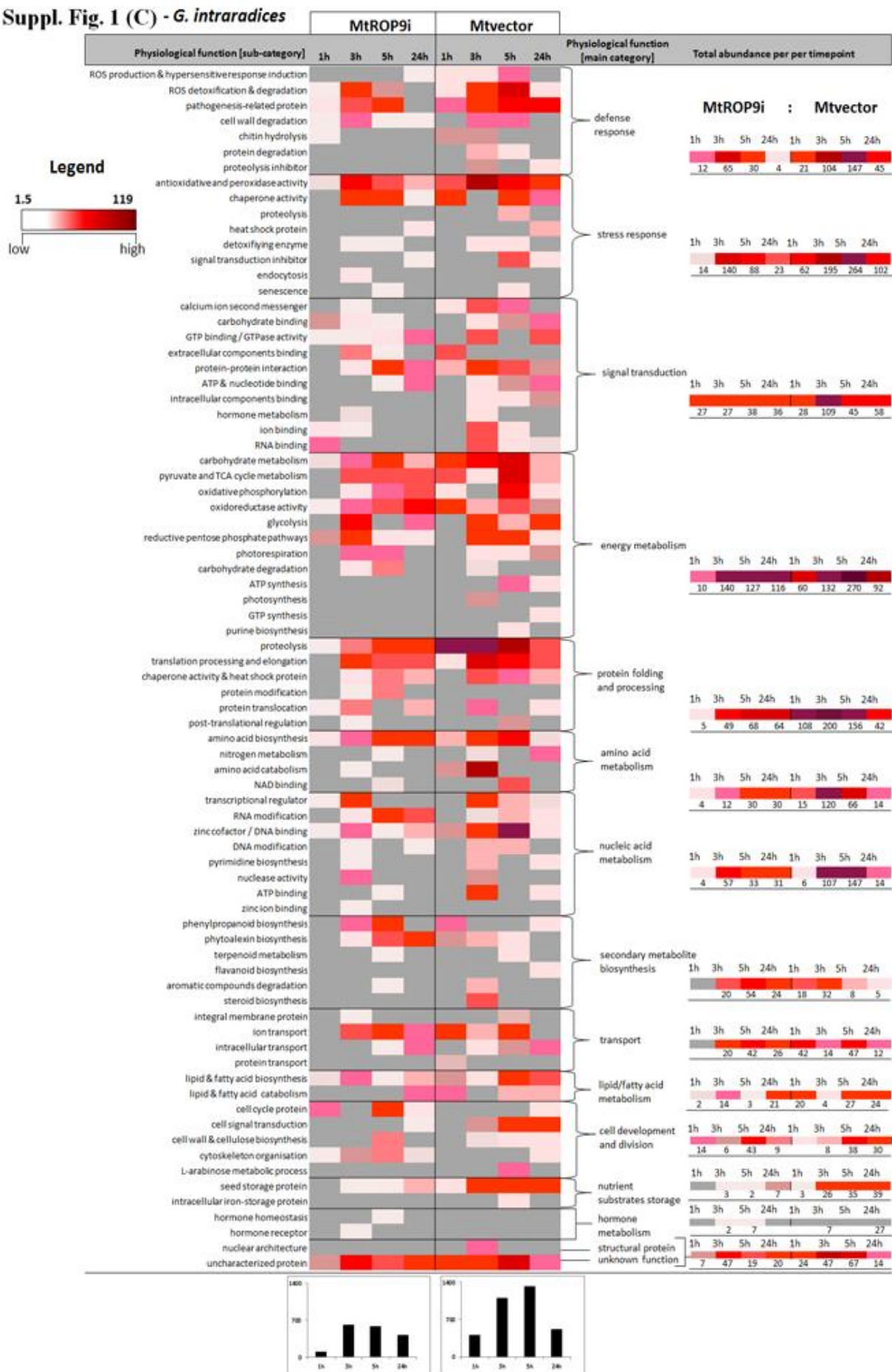

Supplement: Supplementary Figure 1 — Protein induction patterns of M. truncatula MtROP9i and Mtvector roots after S. meliloti (A), A. euteiches (B) and G. intraradices (C) infection visualized on a Heatmap. The heatmap shows distinct proteome subsets created from total induction values of all identified proteins classified into different physiological functions. The color intensity potrayed as a shift in gradient from light red to deep red represents the relative changes from a minimum to a maximum value allowing easy spotting of trends or standouts in each infection at a given time after infection. The total induction for each category is shown on the right and the minigraphs for the trend of total induction over 1, 3, 5, and 24 hpi is shown at the bottom. [file Presentation1.PDF]

Fig. 2 (A) - *S. meliloti*

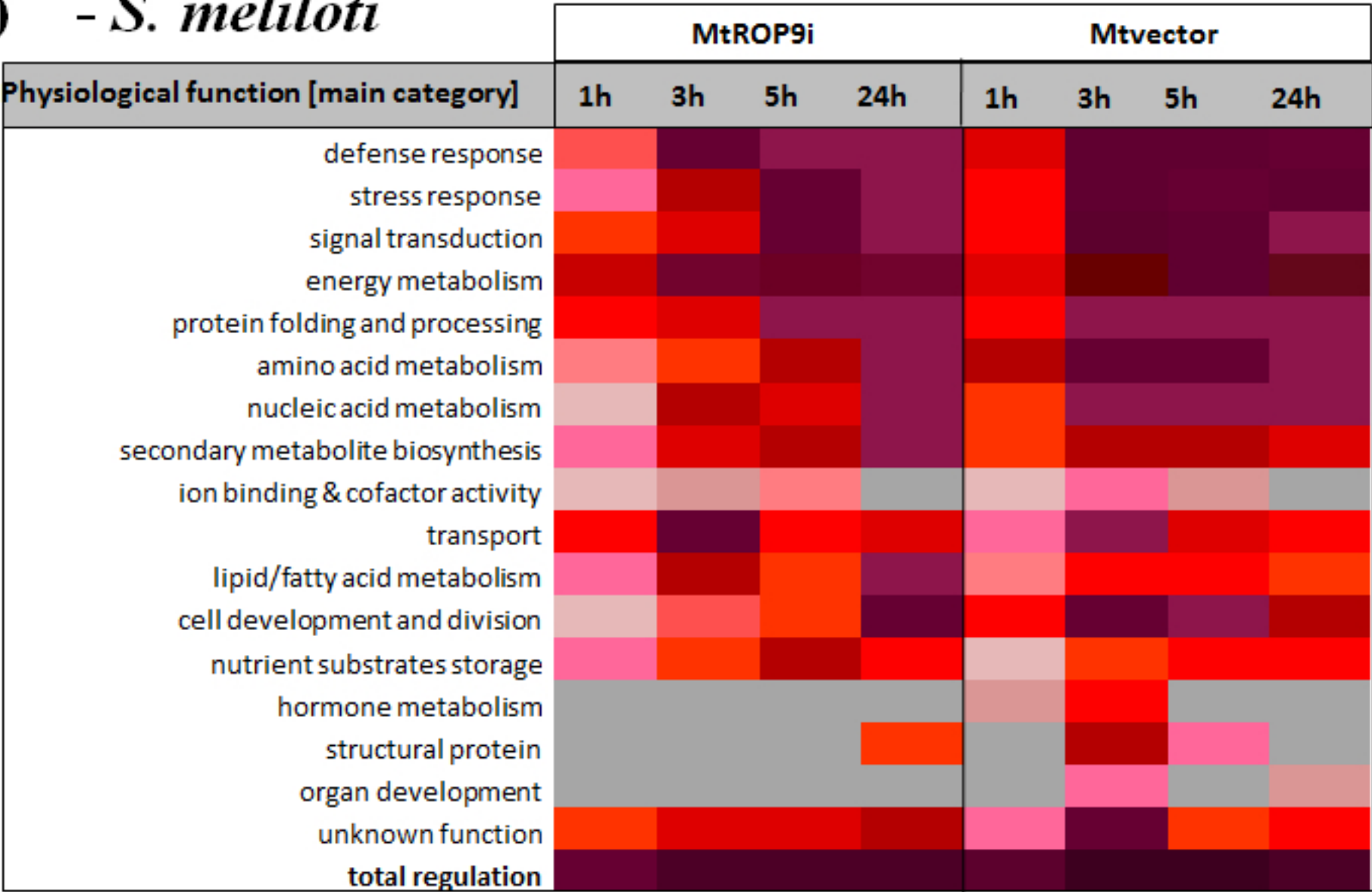

Fig. 2 (B) - *A. euteiches*

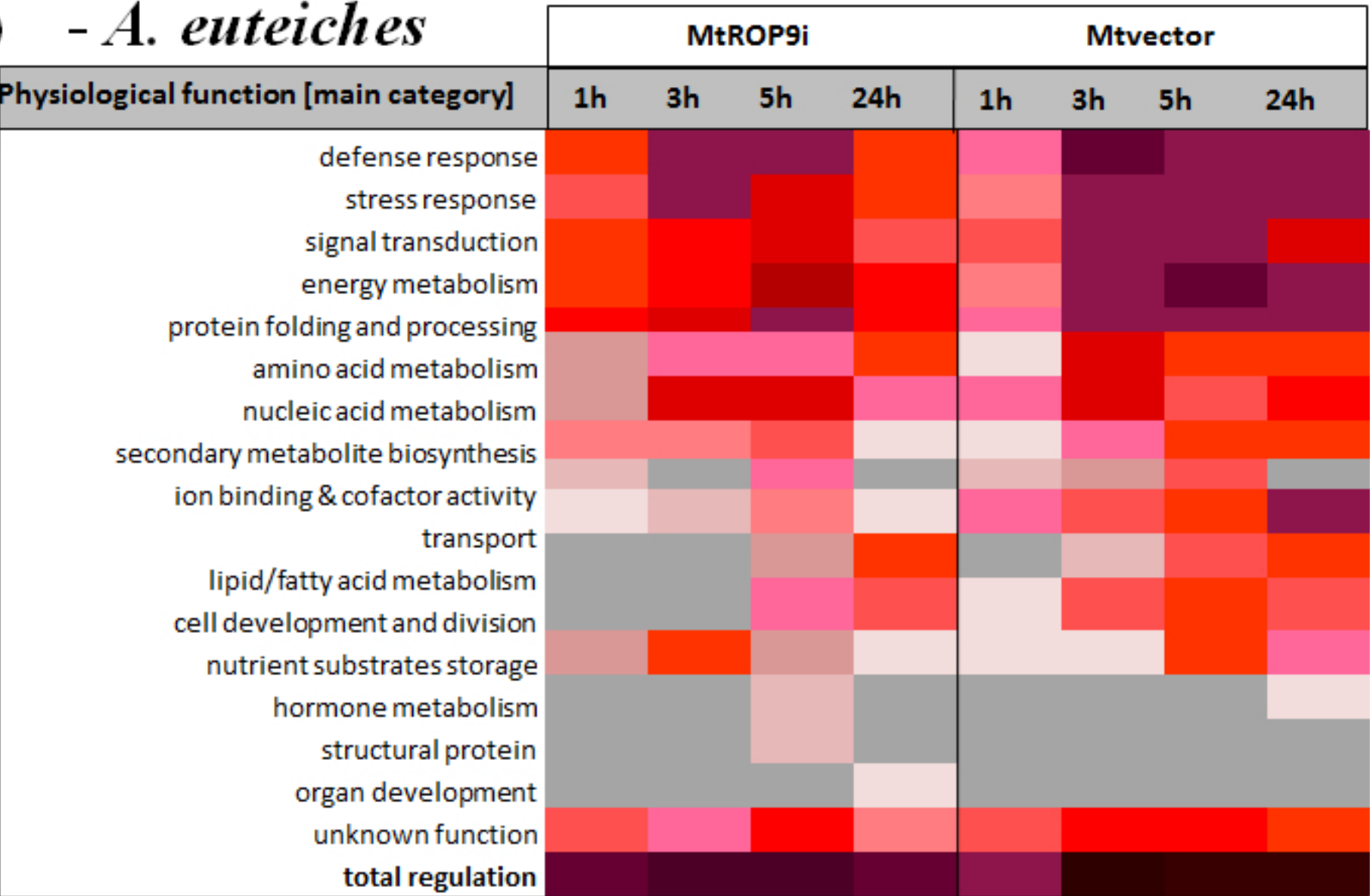

Fig. 2 (C) - *G. intraradices*

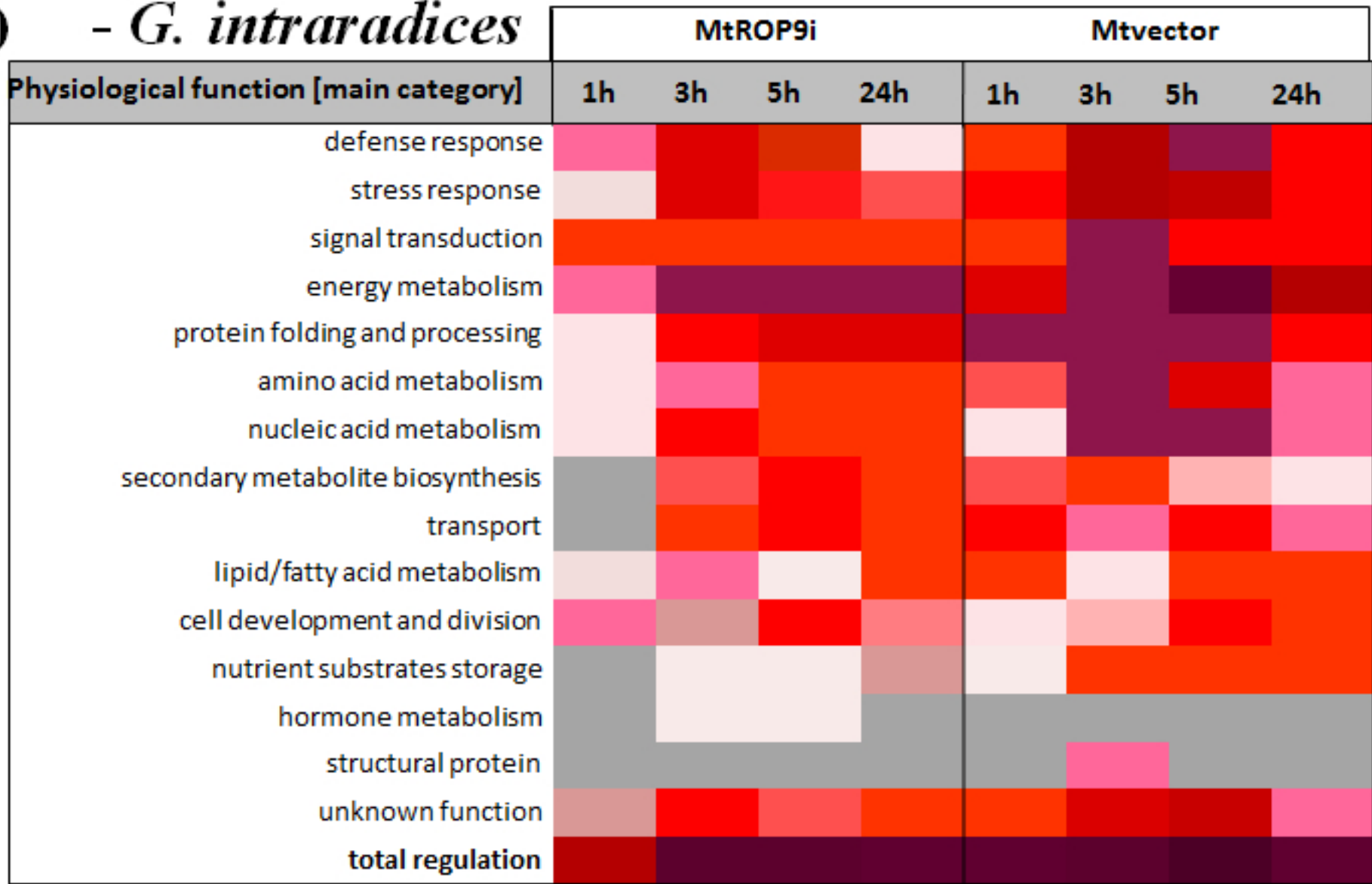

Supplement: Supplementary Figure 2 — Heatmap of total protein regulation in each physiological category over the four time-points for S. meliloti (A), A. euteiches (B) and G. intraradices (C) infections. [file Presentation2.PDF]

**ROP9i**

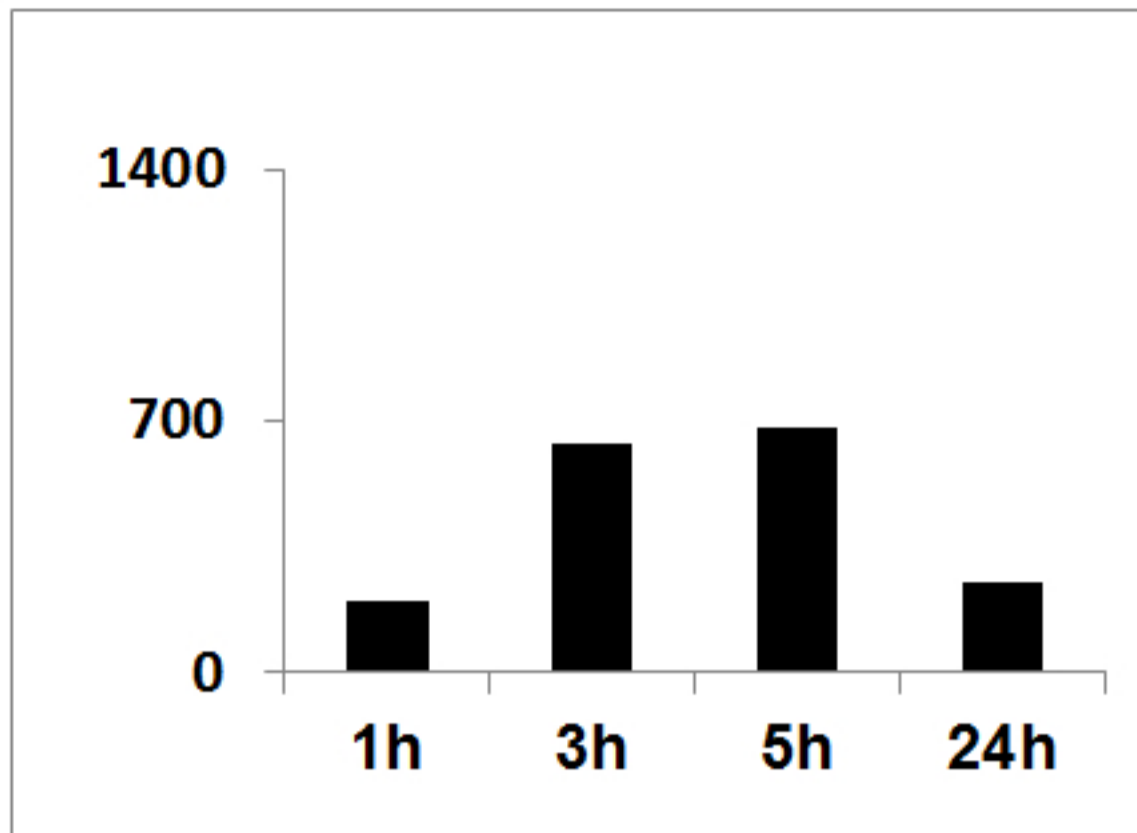

**Mtvector**

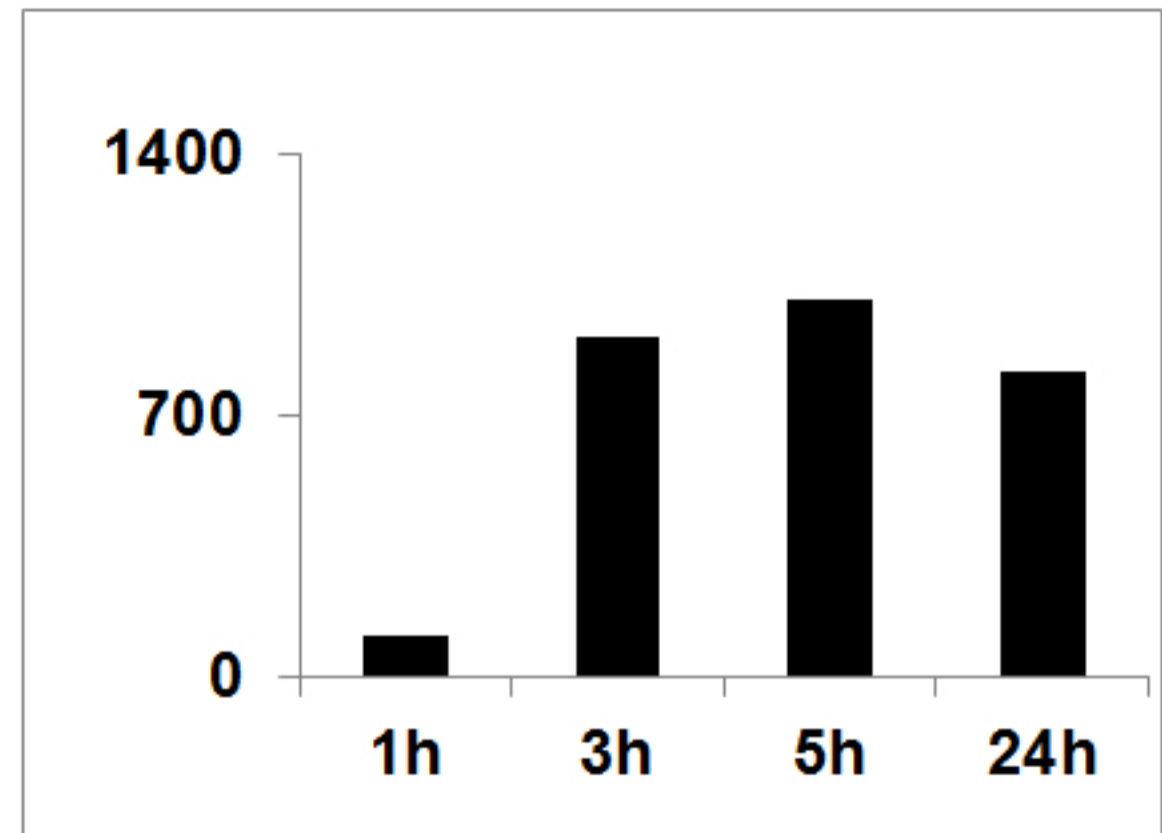

Supplement: Supplementary file 3 [file Presentation3.PDF]
